# Posterior Reversible Encephalopathy Syndrome (PRES) in a Patient With Primary Myelofibrosis on Ruxolitinib

**DOI:** 10.7759/cureus.17944

**Published:** 2021-09-13

**Authors:** Sandhya Shanthosh Kumar, Vignesh Krishnan Nagesh, Keerthana P Sivakolundu, Bahadur Ali, Ibrahim Sange

**Affiliations:** 1 Medicine, Rajarshee Chhatrapati Shahu Maharaj Government Medical College, Kolhapur, IND; 2 Internal Medicine, Stony Brook Southampton, Southampton, USA; 3 Medicine, Government Medical College, Thiruvananthapuram, IND; 4 Medicine, Kilpauk Medical College, Chennai, IND; 5 Medicine, KJ Somaiya Medical College, Mumbai, IND

**Keywords:** hypertension, sepsis, ruxolitinib, primary myelofibrosis, posterior reversible encephalopathy syndrome (pres)

## Abstract

Posterior reversible encephalopathy syndrome (PRES) is a reversible neurological syndrome characterized by headache, seizures, altered mental status, and visual abnormalities, in association with the characteristic bilateral white matter abnormalities in the posterior cerebral hemispheres. As the name suggests, it is typically reversible with clinical recovery within a few days, while the magnetic resonance imaging (MRI) abnormalities resolve much more slowly. We present a 78-year-old female with a known diagnosis of primary myelofibrosis (PMF), on ruxolitinib, a Janus kinase (JAK) 1 and 2 inhibitor, presenting with altered mental status. On presentation, she was hypertensive and with possible sepsis, secondary to urinary tract infection (UTI). She was intubated because of her low Glasgow Coma Scale (GCS), to secure her airways. Computed tomography (CT) of the brain did not reveal any acute ischemic changes. MRI of the brain exhibited findings suggestive of PRES. Ruxolitinib was held and the patient was treated with antihypertensives, anticonvulsants, and antibiotics. Within 24 hours of hospitalization, the patient had a complete neurological recovery, which is diagnostic of PRES. She was extubated successfully and was discharged with a resolution of her symptoms. Although several chemotherapeutic and immunosuppressant drugs are reported to be associated with PRES, the association between ruxolitinib and PRES has not been well established. Thus, case reporting is important to highlight the possible association between ruxolitinib and PRES.

## Introduction

Posterior reversible encephalopathy syndrome (PRES) is a rare neurological disease, more frequently of the posterior subcortical white matter, and was first described in 1996 by Hinchey and colleagues [1]. It predominantly involves the occipital and the parietal lobes, but it can also involve other areas of the brain [2]. The common contributing factors include hypertension (usually severe), kidney disease, severe infections/sepsis, eclampsia, and autoimmune and systemic diseases, as well as immunosuppressants and cytotoxic medications [3].

The presentation is diverse and can include headache, altered mental status, generalized or focal seizures, motor weakness, and visual changes. Diagnosis is made by an MRI of the brain, which identifies and demonstrates areas of vasogenic edema. The treatment is usually targeted toward the removal of the underlying cause contributing to the development of PRES. In most cases, the neurological manifestations are reversible. However, the prognosis depends on the underlying contributing pathology and the neurological sequelae may persist in highly complicated cases [4].

## Case presentation

A 78-year-old female with a history of primary myelofibrosis presented to the emergency department with altered mental status. She was diagnosed with primary myelofibrosis five years ago and has been taking ruxolitinib 5 mg twice daily and monthly darbepoetin infusions for one year.

On presentation, the patient was minimally responsive and history was elicited from her husband. The patient was apparently normal before she experienced a focal seizure in her right hand. Two hours later, the patient was found confused with bowel incontinence and unsteady gait. Her vitals were significant for fever (102.4 Fahrenheit rectal), tachycardia (118 beats/min), tachypnea (28 cycles/min), and elevated blood pressure of 170/76 mmHg. On neurological examination, her Glasgow Coma Scale (GCS) was 6 and another system examination was normal except mild hepatosplenomegaly. Rapid sequence intubation was done due to poor GCS and to protect the airways and the patient was transferred to the ICU.

**Figure 1:**
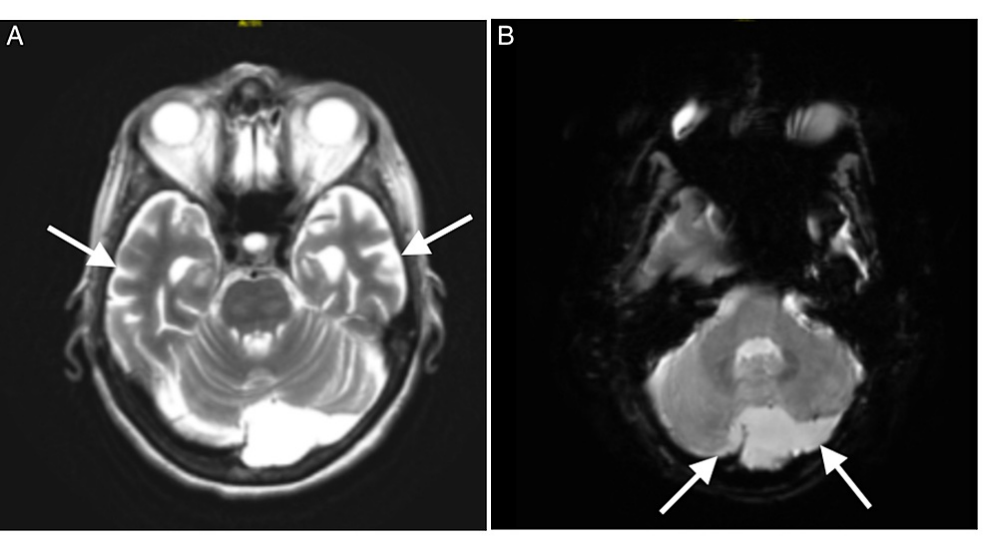
Magnetic resonance imaging of the brain illustrating posterior reversible encephalopathy syndrome in the patient. (A) Fluid-attenuated inversion recovery (FLAIR) sequence signaling bilateral hyperintensity (arrows) involving the temporal regions and extending down to the cerebellar region. (B) Apparent FLAIR hyperintensity in the cerebellar hemispheres (arrows), predominantly on the left.

Her initial laboratory values were remarkable for marked leukocytosis of 32.61 10^3 cells/mm3, anemia with hemoglobin of 10.2 g/dl, hematocrit of 31.7%, and elevated lactate dehydrogenase (LDH) of 669 mg/dl. Blood cultures were sterile. Urine culture was positive for >100,000 *Escherichia coli*. CT of the head revealed moderate involutional and mild microvascular changes as well as a small old lacunar infarct in the right basal ganglia, without any signs of acute ischemia. Electrocardiogram (EKG) initially revealed nonspecific ST-segment changes in inferior and anterolateral leads with elevated troponins. These changes were likely due to the demand-driven ischemia, secondary to her sepsis due to urinary tract infection (UTI). MRI of the brain was significant for scattered areas of subcortical and cortical fluid-attenuated inversion recovery imaging (FLAIR) hyperintensity in the bilateral temporal lobes, with apparent FLAIR hyperintensity in the cerebellar hemispheres as well, consistent with the findings of PRES (Figure 1). Electroencephalogram (EEG) was normal, thus ruling out seizures.

The patient was started on labetalol, prophylactic levetiracetam, as well as broad-spectrum antibiotics (piperacillin-tazobactam). Ruxolitinib was withheld. Eventually, within 24 hours, her mentation improved and she was weaned off the ventilator and extubated. After extubation, she was alert and oriented with no neurological deficits. However, she remained persistently hypertensive, requiring a multidrug regimen with losartan and amlodipine, in addition to labetalol. On day four of hospitalization, she was afebrile, and vital signs and white blood cells count returned to normal. The patient continued to have moderately elevated blood pressure but was otherwise well and was discharged with full recovery two days later. Table 1 summarizes the findings in the patient and the clinical course.

**Table 1:** Patient's characteristics and clinical course. PRES, posterior reversible encephalopathy syndrome.

Variable	Value
Age/sex	78 years/female
Medical history	Primary myelofibrosis
Risk factors for PRES	Ruxolitinib
	Sepsis
	Hypertension
Presentation of PRES	Altered mental status
	Focal seizure
	Bowel incontinence
	Unsteady gait
Vitals	Temperature - 102.4 Fahrenheit (rectal)
	Blood pressure - 170/76 mmHg
	Heart rate - 118 beats per minute
	Respiratory rate - 28 cycles/minute
Glasgow Coma Scale	6
Hemoglobin (gm/dl)	10.2
Hematocrit (%)	31.70
White blood cell count (per mm3)	32.61
Urine culture	Positive for >100,000 colonies of *Escherichia coli*
Treatment administered	Ruxolitinib withheld
	Labetalol
	Losartan
	Amlodipine
	Piperacillin-tazobactam
	Levetiracetam
Duration of hospital stay	6 days
Reason for discharge	Full clinical recovery

## Discussion

Posterior reversible encephalopathy syndrome is a diagnosis of exclusion. Fugate and colleagues proposed a diagnostic criterion, which says that the presence of more than one acute neurological symptom, in addition to the presence of more than one risk factor such as severe hypertension or blood pressure fluctuations, renal failure, immunosuppressant therapy or chemotherapy, eclampsia, autoimmune disorder, with MRI findings suggestive of PRES, warrants the diagnosis of PRES, provided there is no alternate diagnosis that fits into the situation [5].

It is important to identify the underlying triggering factor when PRES is suspected since treatment is generally targeted toward correcting the triggering factor [4]. Chemotherapeutic agents being one of the triggering factors, several of them, as listed in Table 2, have been reported to be responsible for PRES including taxanes, platinum derivatives, vinca alkaloids, antimetabolites, anthracyclines, angiogenesis inhibitors, folate antagonists, and immunosuppressants [6]. However, the exact timeline of initiation of the chemotherapeutic agents and development of PRES has been varied across studies. Laruelle and colleagues reported a case of PRES three months after initiation of sorafenib, a tyrosine kinase inhibitor [7]. The exact mechanism of how these drugs cause PRES is unknown; however, calcineurin inhibitors like tacrolimus and cyclosporine, vascular endothelial growth factor (VEGF) inhibitors such as bevacizumab, and tyrosine kinase inhibitors like sorafenib and sunitinib are known to cause PRES because of their propensity to cause hypertension as their side effect [8].

**Table 2:** Drugs associated with posterior reversible encephalopathy syndrome.

Chemotherapeutic agents	Immunosuppressant drugs
Sorafenib	Methotrexate
Cisplatin	Tacrolimus
Bevacizumab	Sirolimus
Paclitaxel	Rituximab
Vincristine	Ipilimumab
Sunitinib	Cyclosporine
Cytarabine	Interferon alpha
Gemcitabine	Cyclophosphamide

Ruxolitinib is a Janus kinase (JAK) 1 and JAK 2 inhibitor that has been approved in the treatment of myeloproliferative neoplasms associated with progressive bone marrow fibrosis [9]. It is an extremely well-tolerated drug but it is associated with bruising, headaches, dizziness, anemia, and thrombocytopenia, along with an increased risk of infections [10]. However, Sapre and colleagues reported an elevated mean systolic blood pressure in patients taking ruxolitinib [11]. JAK 2 is involved in endothelial, smooth muscle, and cardiac function. JAK 2 inhibition has been reported to impair the expression of endothelial nitric oxide synthase, which might explain the increase in systolic blood pressure secondary to ruxolitinib [11]. Based on these studies, the neurological symptoms due to PRES and new-onset hypertension in our patient may be secondary to the use of ruxolitinib. This can be further supported by the fact that the patient’s clinical symptoms were typically reversed within a day after discontinuation of ruxolitinib.

It is possible that other confounding variables, unrelated to ruxolitinib, like hypertension and sepsis may have led to the development of PRES in our patient. Previous case reports have shown a correlation between control of hypertension and clinical recovery of the patients diagnosed with PRES [12,13]. The persistence of hypertension even after clinical recovery favors the fact that PRES is less likely due to hypertension in this case.

Although previous studies have also established the association between PRES and sepsis, our patient did not have any subjective symptoms of dysuria or fever [14]. In addition, the altered mental status along with focal seizures is more in favor of PRES than delirium secondary to UTI sepsis. Further, the white blood cells (WBC) count in our patient came back to normal four days after receiving antibiotics, but she was clinically stable within a day after discontinuation of ruxolitinib, which also has a short half-life of three hours [9]. These factors suggest that PRES in our patient is most likely secondary to ruxolitinib.

Even though there is a strong association between ruxolitinib and PRES, the treatment targeted all the possible triggering factors like blood pressure control, treatment of sepsis, and discontinuation of ruxolitinib early in the disease course. Early recognition and treatment of PRES are important to facilitate complete neurological recovery.

## Conclusions

The JAK inhibitor ruxolitinib may be associated with the development of PRES in patients with myeloproliferative disorders like primary myelofibrosis (PMF). It is important to consider PRES as one of the differential diagnoses in patients with relevant history presenting with hypertension and neurological symptoms. Identification of the triggering factor leading to the development of PRES is vital, as treatment is targeted toward the elimination of the risk factor.

## References

[1] Hinchey J, Chaves C, Appignani B (1996). A reversible posterior leukoencephalopathy syndrome. N Engl J Med.

[2] Bartynski WS, Boardman JF (2007). Distinct imaging patterns and lesion distribution in posterior reversible encephalopathy syndrome. AJNR Am J Neuroradiol.

[3] Sudulagunta SR, Sodalagunta MB, Kumbhat M, Settikere Nataraju A (2017). Posterior reversible encephalopathy syndrome (PRES). Oxf Med Case Reports.

[4] Fischer M, Schmutzhard E (2017). Posterior reversible encephalopathy syndrome. J Neurol.

[5] Fugate JE, Rabinstein AA (20151). Posterior reversible encephalopathy syndrome: clinical and radiological manifestations, pathophysiology, and outstanding questions. Lancet Neurol.

[6] Patwari A, Bhatlapenumarthi V, Pascual SK (2020). Atypical posterior reversible encephalopathy syndrome due to oral tyrosine kinase inhibitor cabozantinib: first case report. Case Rep Oncol.

[7] Laruelle M, Filleul B, Duprez T, Machiels JP (2018). Posterior reversible encephalopathy syndrome associated with sorafenib and successful retreatment. Urol Int.

[8] Kaur G, Ashraf I, Peck MM, Maram R, Mohamed A, Ochoa Crespo D, Malik BH (2020). Chemotherapy and immunosuppressant therapy-induced posterior reversible encephalopathy syndrome. Cureus.

[9] Arana Yi C, Tam CS, Verstovsek S (2015). Efficacy and safety of ruxolitinib in the treatment of patients with myelofibrosis. Future Oncol.

[10] Galli S, McLornan D, Harrison C (2014). Safety evaluation of ruxolitinib for treating myelofibrosis. Expert Opin Drug Saf.

[11] Sapre M, Tremblay D, Wilck E (2019). Metabolic effects of JAK1/2 inhibition in patients with myeloproliferative neoplasms. Sci Rep.

[12] Abdullah HM, Ullah W, Ahmad E, Anwer F (2016). Posterior reversible encephalopathy syndrome in malignant hypertension secondary to focal segmental glomerulosclerosis. BMJ Case Rep.

[13] Machiraju PK, Alex NM, Safinaaz Safinaaz, Sankaran S (2021). Posterior reversible encephalopathy syndrome as the first manifestation of mixed connective tissue disorder: a case report. J Med Case Rep.

[14] Bartynski WS, Boardman JF, Zeigler ZR, Shadduck RK, Lister J (2006). Posterior reversible encephalopathy syndrome in infection, sepsis, and shock. AJNR Am J Neuroradiol.

